# Self-rated Health and Internet Addiction in Iranian Medical Sciences Students; Prevalence, Risk Factors and Complications

**Published:** 2016-06

**Authors:** Abolfazl Mohammadbeigi, Farzaneh Valizadeh, Seyede Roqaie Mirshojaee, Robabeh Ahmadli, Mohsen Mokhtari, Ebrahim Ghaderi, Ali Ahmadi, Heshmatollah Rezaei, Hossein Ansari

**Affiliations:** 1 Health policy and promotion research center, Department of Epidemiology and biostatistics, School of Health, Qom University of Medical Sciences, Qom,/Iran;; 2 Deputy of Health, Mazandaran University of Medical Sciences, Babolsar, Iran;; 3 MSc Student in health services management, Department of Human Services, Electronic Branch, Islamic Azad University, Tehran, Iran;; 4 Hazrate Masoumeh Hospital, Qom University of Medical Sciences, Qom/Iran;; 5 Health Vic Chancellor, Arak University of Medical Sciences, Arak / Iran;; 6 Social Determinants of Health Research Center, Kurdistan University of Medical Sciences, Sanandaj, Iran;; 7 Modeling in Health Research Center, Department of Epidemiology and Biostatistics, School of Public Health, Shahrekord University of Medical Sciences, Shahrekord, Iran;; 8 Health Promotion research Center, Department of Epidemiology and biostatistics, Zahedan University of Medical Sciences, Zahedan, Iran

**Keywords:** Self-rated health, Internet addiction, risk factors, Students, Iran

## Abstract

**Introduction:**

Self-rated health is a brief measure for general health. It is a comprehensive and sensitive index for prediction of health in future. Due to the high internet usage in medical students, the current study designed to evaluate the self-rated health (SRH) in relationship with internet addiction risk factors in medical students.

**Methods:**

This cross sectional study conducted on 254 students of Qom University of Medical Sciences 2014. Participants selected by two stage sampling method including stratified and simple random sampling. The Young’s questionnaire of internet addiction and SRH question used for data collection. Chi-square, t-test, and logistic regression used in data analysis.

**Results:**

More than 79.9% of students reported their general health good and very good. The student’s mean score of general health was higher than the average. In addition, the prevalence of internet addiction was 28.7%. An inverse significant correlation observed between SRH and internet addiction score (r=-0.198, *p*=0.002). Using internet for Entertainment, using private Email and chat rooms were the most important predictors of affecting to internet addiction. Moreover, internet addiction is the most predictors of SRH and increased the odds of bad SRH.

**Conclusion:**

The good SRH of medical students was higher than general population but in health faculty’ students were lower than others. Due to the effect of internet addiction on SRH and increasing trend of internet use in medical students, as well as low age of participants, attention to psychological aspects and the job expectancy in future, can effective on increasing the good SRH.

## SUMMARY

- The rate of good SRH was 79.9% in medical students and was higher than general population. In addition, the mean score of general health was higher than the average of people.
- The prevalence of internet addiction was 28.7%. Using internet for Entertainment, using private Email and chat rooms were the most important predictors of affecting to internet addiction. Moreover, internet addiction is the most predictors of SRH and increased the odds of bad SRH.
- An inverse significant correlation exist between SRH and internet addiction score.
- It is predicted that poor SRH will increased by higher usage of internet and mobile services and social networks in future. Therefore, preparing cultural and scientific needs for students in leisure times is necessary.

## INTRODUCTION

Self-rated health (SRH) has become as increasing common measure for public health monitoring and has been reported as predictor of mortality in previous studies (1-4). SRH is based on a simple question where people are asked to rate their own overall health (1, 5). It is a subjective consciousness about personal health rather than an objective measure of health. Moreover, SRH has been reported as a predictor of morbidity and mortality even after accounting for objective health status, behavioral risk factors and socio-demographic characteristics and other related factors (5, 6).

Internet addiction is a psychological disorder due to heavy and long usage of internet. An European study showed 68% of people 16-24 age used the Internet compared to 27% of people over age 54 and only 10% of people over 65 (7). However, internet addiction is a common psychological disorder because of technology. Based on recent study the prevalence of internet addiction in Iranian students is 10.8% (8) and related to some adverse outcome including depression and suicide in students (9).

Today, undergraduate university students contain a large portion of the younger population in Iran (10) and an increasing number of university students are earning their degrees online (11). They will have a considerable influence in society through the key roles adopted in the future as professionals, senior executives, and politicians. Thus far, the current study aimed to find the relationship between SRH, internet addiction, and determinants of SRH and internet addiction among university students that have not been studied to now.

## MATERIAL AND METHODS

A cross sectional study conducted on 254 students in Qom Medical University those were in second term or higher. Subjects selected by two stage sampling method including stratified proportional sampling in the first stage and faculty considered as stratum. In the second stage, simple random sampling was used to all students have equal chance for selection.

Data collection conducted by standard questionnaires including demographic characteristics, Young internet addiction questionnaire (YIAQ) and SRH question.

### Young internet addiction questionnaire (YIAQ)

This questionnaire has 20 items in Likert scale and the reliability and validity of YIAQ is measured and validated by several studies in Iran (8, 12, 13). In addition , the Persian version of questionnaire is validated by backward-forward translation method and published in our recent article (12). Also, the reliability of Persian YIAQ calculated 0.917 (12). Respondents should select one of the alternatives in each items from five point likert’ scale varied from rarely; 1) occasionally; 2) frequently; 3) often; 4) always; 5) The sum of scores of items calculates the internet addiction score and the higher score shows greater level of internet addiction. The scores varied between 20 to 100 and categorized in three level including 20 -39 as online users with complete control on usage, 40 -69 as users with frequent problems due to internet usage and 70-100 as sever addiction that internet is causing significant problems (12, 14, 15).

### SRH

The SRH measured by tree different questions with likert scale. 1) SRH-5; how would you rate your general health status? With reply alternatives: very good, quite good, neither good nor poor, quite poor, and poor; 2) SRH-7; how do you regard your health? With reply alternatives ranging from 1 to 7, where 1 = Very poor, and 7 = Excellent, could not be better; 3) SRH-age; how would you assess your general health status compared to that of others of your own age? With reply alternatives: Much better, slightly better, neither better nor worse, slightly worse and much worse. The reliability and validity of these questions are assessed in other studies (1, 16, 17).

Data entered in SPSS software and were analyzed by chi-square, t-test analysis of variance, and Spearman correlation coefficient tests. Rating health as very good to very poor seems a simple concept that is unlikely to contain surprises, but ratings of health as fair or poor are predictors of morbidity and mortality after adjusting for clinical health status (18). Therefore, logistic regression used after recoding very good and quite good considered as good SRH and other alternatives as poor SRH. Ethical committee of Qom University of Medical Sciences approves the study protocol. In addition, informed consent filled by all subject before enrolment.

## RESULTS

Overall, 254 medical students were studied with mean age of 21.7 ± 2.9 years which 84.3% (214 students) were female and 77.6% (197 students) were single. From total, 36.6% (93 students) resident in dormitory and 89.8% have not any health problem or disease history.

More than 81.9% reported their self-health as good quietly (50%) or very good (31.9) and only 3.2% were poor quietly or very poor. Other students (14.9%) reported as moderate. In addition, 41.5% of students reported their health status as the same of other coeval while in 12% were worse than and in 46% were better than ones. The mean of three different SRH questions are depicted in Table 1. The mean score for three SRH question were significantly higher the mean. Chi square test (Table 2) showed that faculty, using email and scientific websites and having scientific research are related with good self-rated health while internet addiction is associated with bad self-rated health. There were not significant relationship between SRH and marital status, gender, using chat room and being in dormitories of university. It is considerable that the lowest SRH reported in students of health faculty. In addition, t-test showed that there was a significant association between SRH and age of subjects. In a way, that higher age was related with worse SRH. However, educational average score, educational term, work with computer, and work with internet were not related with SRH status.

**Table 1:** Minimum, Maximum, Mean± standard deviation of self-rated health score by three different approach

Scale	Minimum	Maximum	Mean ± SD	Difference with mean score	*P* value

Total General Health	1	5	4.1 ± 0.798	1.6	<0.001
GH compared to coeval	1	5	3.55 ± 0.98	1.05	<0.001
GH from 7	1	7	5.23 ± 1.06	1.73	<0.001

**Table 2:** Comparing the demographic factors in participants according to self-rated health status

	Good self-rated n=200 n (%)	Bad self-rated n=45 n (%)	

Using from Email	168 (84)	32 (71.1)	0.04
Using Chat room	46 (23.1)	11 (25)	0.463
Internet addicted	50 (24.6)	22 (48.9)	<0.001
Female sex	171 (84.7)	38 (84.4)	0.563
Single marital	45 (22.2)	10 (22.2)	0.566
Being in dormitory	72 (35.6)	20 (46.5)	0.123
Using scientific webs	79 (38.9)	8 (17.8)	0.005
Having scientific research	81 (41.5)	93 (26.7)	0.045
Faculty of Medical	42 (93.3)	3 (6.7)	0.016
Faculty of Paramedical	37 (82.2)	8 (17.8)	
Faculty of Nursing	32 (91.4)	3 (8.6)	
Faculty of Health	92 (74.8)	31 (25.2)	
Educational average score	16.72 ± 1.59	16.6 ± 1.55	0.717
Educational term	5.69 ± 1.8	5.74 ± 1.88	0.879
Age	21.43 ± 2.29	22.54 ± 4.27	0.013
Work with computer	10.98 ± 11.7	9.57 ± 10.14	0.458
Work with internet	7.02 ± 8.75	7.12±9.24	0.590

The mean score of internet addiction was 35.22 ± 12.1 and subjects weekly used on average 10.5 ± 11.35 and 7.6 ± 8.8 hours by PC (private computer) and internet, respectively. Moreover, 71.3% were normal regarding to internet use. The prevalence of internet addiction was 28.7% including 27.2% mild internet addiction and 1.6% were severe addicted with frequent problems due to internet. According to Chi-square test (Table 3) female gender, using internet for entertainment, using chat rooms, and have Email are associated by affecting to internet addiction. In addition, t-test showed that internet addiction is related to decrease in education score average, higher usage of internet and PC. Moreover, an inverse significant correlation observed between SRH and internet addiction score (r=-0.198, *p*=0.002). Nevertheless, marital status, living in dormitory, educational level, conducting scientific research, and having Lap Top were not significant factors.

**Table 3:** Comparing the demographic factors in participants according to Internet addiction

Variables	Normal	Internet addicted	*P* value

Age	21.8 ± 2.97	21.49 ± 2.65	0.467
Average score	16.9 ± 1.45	16.27 ± 1.8	0.01
Work with PC (hours/week)	7.99 ± 7.73	16.8 ± 16.7	<0.001
Work in Internet (hours/week)	5.01 ± 5.6	13.9 ± 11.4	<0.001
Male gender	23 (12.7)	16 (22.2)	**0.047**
Being single	136 (75.1)	61 (83.6)	0.097
Living in dormitory	63 (35)	30 (42.3)	0.177
Conditioned pass	6 (3.4)	5 (6.8)	0.186
Bachelor level	149 (82.8)	54 (74)	0.261
Entertainment	72 (39.8)	46 (63)	**0.001**
News	46 (25.4)	22 (30.1)	0.268
Scientific Search	123 (68)	41 (56.2)	0.052
Academic research	71 (40.8)	23 (32.4)	0.139
Owning Lap Top	60 (33.7)	27 (37.5)	0.334
Private Email	138 (77.5)	67 (93.1)	**0.002**
Chat room Using	25 (14)	34 (48.6)	**<0.001**

Finally, logistic regression model showed that using internet for entertainment, using private Email and chat rooms were the most important predictors of affecting to internet addiction disorder. So that, using chat room increase the odds of internet addiction near 5 fold (OR=4.93, CI 0.95: 2.54-9.6). Also, using Email increase to three fold (OR=3.11, CI 0.95: 1.02-9.4) and using internet for entertainment increase to 2.2 fold (OR=2.22, CI 0.95: 1.2-4.23) the odds of affecting to internet addiction. Moreover, internet addiction was the most predictors of SRH so that increase the odds of bad SRH more than 3 fold (OR=3.01, CI 0.95: 1.4-6.3).

## DISCUSSION

In the current study, poor and moderate response to SRH was 18.1%. in another study conducted by Babones this rate was 8.3% (17). In a national survey in Iran 54% of studies subjects reported the SRH as good and their SRH score were 2.16 (19). In the current study, the mean score of SRH was 4.1 and 3.55 in comparing to their coevals. Differences among studies are related to different age of study population and different morbidity and general health status. It is well established that age is one of the most important factors of SRH. Moreover, our results showed that by increasing in age the good response to SRH is decreased. Sulander study is confirmed this results (20). A longitudinal Australian study conducted in 2001 and 2004 showed that the very good and excellent SRH increase from 52% to 56% (21). In Vahdania study the population age were between 18 to 65 years old and with the mean age was 32.7 years and 71% reported their SRH, as good and very good and only 8% were very bad. That study showed that socioeconomic status, income level, job and living city were related to SRH (19). There was no relationship between good SRH and educational level and gender of students. According to other studies, socioeconomic status and income level is related to health care utilization and better perception about general health (22, 23). However, in Sulander *et al.* study lower literature was related by poor SRH (20). In addition, studies showed that lonely male and with lower income are susceptible for week SRH (4, 24-26).

The prevalence of internet addiction was 28.7% and this prevalence was higher than other studies conducted in Iran (8), Norwegian (27) and Korean (28) students. However, based on the results of studies depression and anxiety are related with internet addiction and caused by some problems in educational course (9, 29).

According to our results using internet for entertainment, using from chat rooms and private Email are predictors of internet addiction. In addition, internet addiction is the most important predictors of SRH in medical students. Another study showed the effect of internet use on SRH (7). According to results of a recent study biological indicators are related to SRH (30). It is showed that affecting to any disease as well as internet addiction is associated with SRH. However, it is possible that potential relationships between Internet use and health might be as a result of relationship between socioeconomic status or determinant of access and use of the internet with health rather than effects of internet use by itself (7) Also, according to other studies poor SRH is powerfully and independently associated with somatization (18). However, high usage of chat rooms and Email is effective on sleep quality (5) and individuals with negative SRH were with higher affected to anxiety (18).

However, since these relationships accessed in a cross-sectional study the temporality of association is unclear. Therefore, authors suggested that prospective studies conducted in general population in different age groups based on psychological outcomes and common disease to find the effect on SRH as predictors of health.

## CONCLUSION

The rate of good SRH in Qom medical university was higher than general population but low in comparing to their age. Poorer SRH was observed in students of health faculty. Since the target population of our study was young, the poor response of SRH in medical students is considerable. It is predicted that this prevalence will increased by higher usage of internet and mobile services and social networks in future. Therefore, medical universities shall to prepare the cultural and scientific needs of students in leisure times. Therefore, increasing self-steam, insured, and clear future can remove fear and anxiety and help for increasing of student is SRH.

## References

[1] Eriksson I, Undén A-L, Elofsson S, Self-rated health (2001). Comparisons between three different measures. Results from a population study. International Journal of Epidemiology.

[2] Burstrom B (2012). Commentary: self-rated health and mortality in low income settings. Int. J. Epidemiol.

[3] Benyamini Y, Blumstein T, Lusky A, Modan B (2003). Gender differences in the self-rated health–mortality association: Is it poor self-rated health that predicts mortality or excellent self-rated health that predicts survival?. The Gerontologist.

[4] Zheng H, Thomas PA (2013). Marital status, self-rated health, and mortality: overestimation of health or diminishing protection of marriage?. J. Health Soc. Behav.

[5] Frange C, de Queiroz SS, da Silva Prado JM, Tufik S (2014). The impact of sleep duration on self-rated health. Sleep Science.

[6] Shin HY, Shin MH, Rhee JA (2012). Gender differences in the association between self-rated health and hypertension in a Korean adult population. BMC Public Health.

[7] Gracia E, Herrero J (2009). Internet Use and Self-Rated Health Among Older People: A National Survey. J. Med. Internet Res.

[8] Ghamari F, Mohammadbeigi A, Mohammadsalehi N, Hashiani AA (2011). Internet addiction and modeling its risk factors in medical students, iran. Indian journal of psychological medicine.

[9] Kim K, Ryu E, Chon M-Y, Yeun E-J (2006). Internet addiction in Korean adolescents and its relation to depression and suicidal ideation: a questionnaire survey. International journal of nursing studies.

[10] (2012). Statistical Center of Iran. General Population and Housing Census 2006. http://www.google.com/url?sa=t&rct=j&q=&esrc=s&frm=1&source=web&cd=1&cad=rja&ved=0CDEQFjAA&url=http%3A%2F%2Fwww.amar.org.ir%2FPortals%2F2%2Fcensus-90%2Fjamiat%2FKol%2FKol-Jamiat-03.xls&ei=E2AzUZbgOubk4QTdk4Bw&usg=AFQjCNEepNYTlIcTeNqyL0LR9MCKwf9XGQ&sig2=1gYsBN3bMpg7Ccakj0Crdg.

[11] Rohrer J, Cole L, Schulze F (2012). Cigarettes and Self-Rated Health Among Online University Students. J. Immigrant Minority Health.

[12] Mohammadsalehi N, mohmmadbeigi A, Jadidi R, Anbari Z (2015). Psychometric Properties of Persian Language Version of Yang Internet Addiction Questionnaire; an Explanatory Factor Analysis. International Journal of High Risk Behaviors and Addiction.

[13] Dargahi H, Razavi S (2007). Internet addiction and its related factors: a study of an Iranian Population. Payesh.

[14] Widyanto L, McMurran M (2004). The psychometric properties of the internet addiction test. Cyberpsychol Behav.

[15] Young KS (2000). Internet addiction: The emergence of a new clinical disorder. CyberPsychology & Behavior.

[16] Chandola T, Jenkinson C (2000). Validating self-rated health in different ethnic groups. Ethnicity and Health.

[17] Babones SJ (2009). The consistency of self-rated health in comparative perspective. Public Health.

[18] Mewton L, Andrews G (2013). Poor self-rated health and its associations with somatisation in two Australian national surveys. BMJ Open.

[19] Vahdania M, Ebadi M, Azin A, AEENPARAST A (2011). How people rate their own health: a nationwide study from Iran. Payesh.

[20] Sulander T, Pohjolainen P, Karvinen E (2012). Self-rated health (SRH) and socioeconomic position (SEP) among urban home-dwelling older adults. Archives of gerontology and geriatrics.

[21] Greenacre M (2002). Correspondence analysis of the Spanish national health survey. Gaceta Sanitaria.

[22] Mohammadbeigi A, Hassanzadeh J, Eshrati B, Rezaianzadeh A (2013). Socioeconomic inequity in health care utilization, Iran. Journal of epidemiology and global health.

[23] Kavosi Z, Mohammadbeigi A, Ramezani-Doroh V, Hatam N, Jafari A, Firoozjahantighi A (2015). Horizontal inequity in access to outpatient services among Shiraz City residents, Iran. Journal of research in health sciences.

[24] Brunner RL (2006). Understanding gender factors affecting self-rated health. Gender Medicine.

[25] Pilcher JJ, Ginter DR, Sadowsky B (1997). Sleep quality versus sleep quantity: relationships between sleep and measures of health, well-being and sleepiness in college students. Journal of psychosomatic research.

[26] Sirola J, Tuppurainen M, Rikkonen T, Honkanen R, Koivumaa-Honkanen H, Kröger H (2010). Correlates and predictors of self-rated health and ambulatory status among elderly women - Cross-sectional and 10 years population-based cohort study. Maturitas.

[27] Johansson A, Götestam KG (2004). Internet addiction: characteristics of a questionnaire and prevalence in Norwegian youth (12–18 years). Scandinavian journal of psychology.

[28] June KJ, Sohn SY, So AY, Yi GM (2007). A study of factors that influence Internet addiction, smoking, and drinking in high school students. Journal of Korean Academy of Nursing.

[29] Mohamadbeigi A, Ghazavi A, Ghamari F, Saeidi A (2010). Effect of internet addiction on educational status of Arak University of medical sciences students, spring 2009. Arak Medical University Journal.

[30] Jylhä M, Volpato S, Guralnik JM (2006). Self-rated health showed a graded association with frequently used biomarkers in a large population sample. Journal of Clinical Epidemiology.

